# Inhaled prostacyclin therapy in the acute respiratory distress syndrome: a randomized controlled multicenter trial

**DOI:** 10.1186/s12931-023-02346-0

**Published:** 2023-02-18

**Authors:** Helene A. Haeberle, Stefanie Calov, Peter Martus, Lina Maria Serna-Higuita, Michael Koeppen, Almuth Goll, Alice Bernard, Alexander Zarbock, Melanie Meersch, Raphael Weiss, Martin Mehrländer, Gernot Marx, Christian Putensen, Tamam Bakchoul, Harry Magunia, Bernhard Nieswandt, Valbona Mirakaj, Peter Rosenberger

**Affiliations:** 1 Department of Anesthesiology and Intensive Care Medicine, Tübingen University Hospital, Hoppe-Seyler-Straße 3, 72076 Tübingen, Germany; 2 Institute for Clinical Epidemiology and Applied Biometry, Faculty of Medicine, University of Tübingen, Tübingen, Germany; 3 Department of Anesthesiology, Intensive Care and Pain Medicine, University of Münster, Münster, Germany; 4 Department of Intensive Care Medicine, University Hospital RWTH Aachen, Aachen, Germany; 5 Department of Anesthesiology and Intensive Care Medicine, University Hospital Bonn, Bonn, Germany; 6 Institute of Experimental Biomedicine I, University Hospital Würzburg, Würzburg, Germany; 7 Transfusion Medicine, Medical Faculty of Tuebingen, University Hospital of Tuebingen, Tübingen, Germany

**Keywords:** ARDS, Prostacyclin, COVID-19, Inflammation, Therapy

## Abstract

**Background:**

Acute respiratory distress syndrome (ARDS) results in significant hypoxia, and ARDS is the central pathology of COVID-19. Inhaled prostacyclin has been proposed as a therapy for ARDS, but data regarding its role in this syndrome are unavailable. Therefore, we investigated whether inhaled prostacyclin would affect the oxygenation and survival of patients suffering from ARDS.

**Methods:**

We performed a prospective randomized controlled single-blind multicenter trial across Germany. The trial was conducted from March 2019 with final follow-up on 12th of August 2021. Patients with moderate to severe ARDS were included and randomized to receive either inhaled prostacyclin (3 times/day for 5 days) or sodium chloride (Placebo). The primary outcome was the oxygenation index in the intervention and control groups on Day 5 of therapy. Secondary outcomes were mortality, secondary organ failure, disease severity and adverse events.

**Results:**

Of 707 patients approached 150 patients were randomized to receive inhaled prostacyclin (n = 73) or sodium chloride (n = 77). Data from 144 patients were analyzed. The baseline PaO_2_/FiO_2_ ratio did not differ between groups. The primary analysis of the study was negative, and prostacyclin improved oxygenation by 20 mmHg more than Placebo (p = 0.17). Secondary analysis showed that the oxygenation was significantly improved in patients with ARDS who were COVID-19-positive (34 mmHg, p = 0.04). Mortality did not differ between groups. Secondary organ failure and adverse events were similar in the intervention and control groups.

**Conclusions:**

The primary result of our study was negative. Our data suggest that inhaled prostacyclin might be beneficial treatment in patients with COVID-19 induced ARDS.

*Trial registration:* The study was approved by the Institutional Review Board of the Research Ethics Committee of the University of Tübingen (899/2018AMG1) and the corresponding ethical review boards of all participating centers. The trial was also approved by the Federal Institute for Drugs and Medical Devices (BfArM, EudraCT No. 2016003168-37) and registered at clinicaltrials.gov (NCT03111212) on April 6th 2017.

**Supplementary Information:**

The online version contains supplementary material available at 10.1186/s12931-023-02346-0.

## Introduction

Acute respiratory distress syndrome (ARDS) is a common, life-threatening syndrome characterized by the development of severe hypoxia. The hallmark of SARS-CoV-2 infection is COVID-19-induced ARDS, which is associated with severe hypoxia. This hypoxia affects the function of secondary organs, and as a result, organ failure in the affected tissues may develop [1]. The underlying cause of ARDS is uncontrolled and self-propagating inflammation within the alveolar space associated with the loss of pulmonary barrier function [2]. Several pharmacological approaches have been tested in the past to improve oxygenation and overall outcomes of patients with ARDS with varying results [3–5].

Prostacyclins are used to treat patients with dyspnea due to pulmonary arterial hypertension, which is often associated with endothelial changes within the pulmonary vasculature [6, 7]. ARDS, particularly COVID-19-induced ARDS, is characterized by pathological features such as endothelial injury, suggesting that prostacyclin therapy might be beneficial [8]. A small, single-center observational study suggested that prostacyclins might improve oxygenation in patients suffering from ARDS [9]. In COVID-19 ARDS the infusion of prostacyclin was not associated with a significant reduction of mortality and did not increase the number of days alive. A point estimate analysis however done after the end of the trial favored the prostacyclin group [10]. However, to date no systematic investigations have evaluated the effect of inhaled prostacyclin on a population suffering from ARDS. The aim of this trial was to test the hypothesis that prostacyclin would improve oxygenation and clinical outcomes of patients with ARDS, regardless of its cause [11].

## Methods

### Study design, ethics and oversight

We conducted a prospective randomized controlled, single-blind multicenter trial administering prostacyclin to critically ill patients with ARDS for 5 days. Two major changes in the design were amended in the protocol. First, patients who did not receive the study therapy according to the physician’s decision were included in the primary analysis population to avoid bias. Second, an extensive subgroup analysis was performed for patients with COVID-19, as the pandemic started during the study period. The study was approved by the Institutional Review Board of the Research Ethics Committee of the University of Tübingen (899/2018AMG1) and the corresponding ethical review boards of all participating centers. The trial was also approved by the Federal Institute for Drugs and Medical Devices (BfArM, EudraCT No. 2016003168-37) and registered at clinicaltrials.gov (NCT03111212). For further details, please see Additional file 1. ARDS aetiologies, such as viral or bacterial infection, were diagnosed by routine laboratory diagnostic tests within the participating institutions.

### Patients

Before the inclusion of patients into the study, the trial coordinators obtained consent for participation in the study. Only patients older than 18 years were allowed to enter the study. All patients received echocardiography to exclude right ventricular failure or pulmonary hypertension. For details about inclusion and exclusion criteria please see Additional file 1.

### Randomization and interventions

Randomization was performed at a 1:1 ratio using a parallel group design. Randomization lists were generated at the biostatistical center using the software nQuery, release 4, and based on these lists, numbered envelopes were provided and used for randomization (stratified for center and using blocks of random length). For each center, a separate spate list was generated, and closed envelopes were supplied to the participating centers. Envelopes were opened only by the treating physician. The randomization number and treatment were recorded in the ID screening and enrollment list, dated and signed. The signed sheet was then stored at the participating center. Random treatment allocation was used to protect against selection bias. Concealment bias was not present, because the person who was recruiting patients was informed after recruitment about the assigned study arm. The primary and secondary endpoints were objectively measurable, which excluded information bias. Intervention was inhalation with Iloprost (20 µg/3times per day in 10 ml NaCl 0.9% for 5 days) or inhalation of NaCl 0.9% (10 ml) as Placebo [11].

### Outcomes

The primary endpoint was the improvement in oxygenation defined as the PaO_2_/FiO_2_ ratio on Day 5 of therapy. This outcome should not be affected by observation bias, as it is based on an objective routine measurement. Secondary outcomes included overall survival in the 90-day follow-up period; SOFA Organ Failure (SOFA) scores on Days 1–14, 28 and 90; duration of mechanical ventilation support; ICU length of stay; development of ventilator-associated pneumonia, pulmonary hemorrhage, gastrointestinal hemorrhage, pulmonary embolism, coagulopathy, delirium, ICU-acquired weakness and discharge location.

### Sample size

In a previous study of prostacyclin effect in 20 patients, an increase from 177 ± 60 mmHg to 213 ± 67 mmHg was observed for PaO_2_/FiO_2,_ which was significant at the 0.01 level in an intraindividual comparison [9]. Recalculation showed that the standard deviation was considerably smaller, as a p value of 0.01 corresponds to an effect size of 0·93 (intraindividual) and thus to an intraindividual standard deviation of approximately 40 in this study. For details about sample size see Additional file 1.

### Statistical analysis

**Fig. 1:**
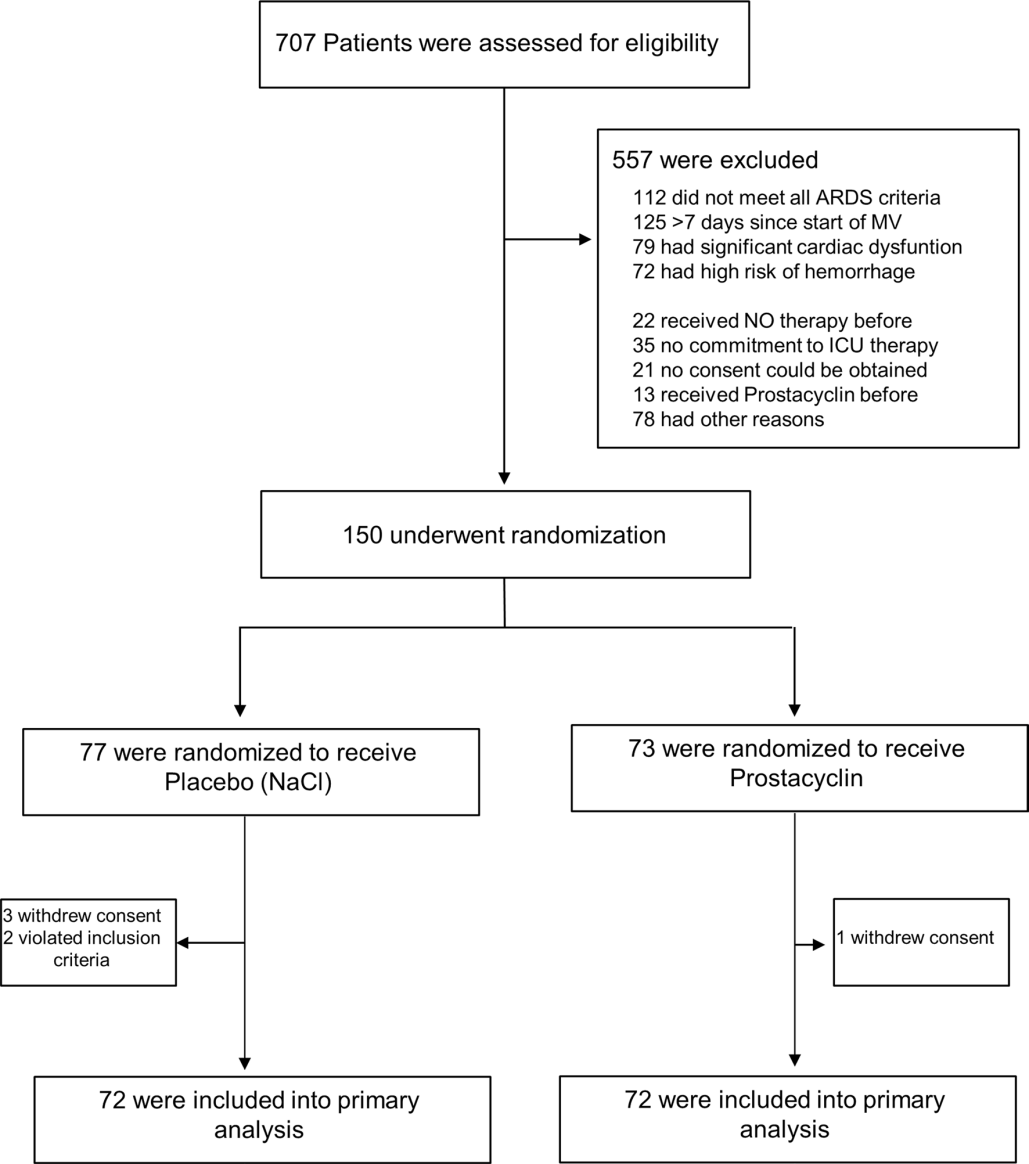
Enrollment and Randomization of Patients

The primary hypothesis of the analysis was to show the superiority of inhaled prostacyclin to placebo (NaCl). The primary analysis population was the intention to treat the population of randomized patients and provide baseline values, except for six patients who were excluded for reasons documented in the Consort Flowchart (Fig. 1). The primary endpoint, Pa_a_O_2_/FiO_2_, on Day 6 after baseline, i.e., Day 5 of prostacyclin treatment, was evaluated using a baseline-adjusted analysis of covariance model with the last measurement of Pa_a_O_2_/FiO_2_ before treatment serving as the baseline and the study arm and center as two-level factors. For further details see Additional file 1.

## Results

### Enrollment and patients

The trial was conducted from March 2019 to August 2021. Seven hundred seven patients were screened for inclusion, of whom 150 patients were enrolled and randomized to receive either placebo l or prostacyclin (Iloprost®) inhalation 3 times/day for 5 days (Fig. 1). The last patient was recruited on 14.05.21, and 144 patients were included in the primary analysis (n = 72 placebo, n = 72 prostacyclin) since 6 patients withdrew consent during the course of the trial or during the observation period (n = 4) or violated the inclusion criteria (n = 2) (Fig. 1). The baseline characteristics of the patients are presented in Table 1. These characteristics were similar in both study groups (Table 1). The age of the intervention group was significantly higher than that of the control group at 61.5 years compared to 58.5 years. Regarding the pre-existing comorbidities, the group of patients treated with prostacyclin showed a higher incidence of pre-existing COPD and emphysema. The main causes of ARDS were COVID-19-induced ARDS, followed by bacterial infection that resulted in ARDS. Organ specific baseline characteristics and ventilation parameters did not differ between groups. There were more patients receiving extracorporeal membrane oxygenation (ECMO) therapy in the Placebo group than in the prostacyclin group (21 vs. 15), yet this difference was not significant (Additional file 1: Table S1). With regard to the therapies used both groups did not differ significantly with regards to steroid therapy, the use of IL-6 antibodies or the treatment with remdesivir (Table 1). These therapies were used exclusively in COVID-19 patients. Neuromuscular blockers were not used at all in the study population. Prone positioning was used in both study groups according to ARDS guidelines [12].

**Table 1:** Demographic and baseline characteristics

	Control (n = 72)	Prostacyclin (n = 72)
Age, mean ± SD, years	56.0 ± 14.0	61.1 ± 14.4*
Weight, mean ± SD, kg	93.6 ± 20.7	93.3 ± 23.8
Height, mean ± SD, cm^a^	174.4 ± 9.2	174.4 ± 9.2
Body Mass Index^a^	30.8 ± 6.5	30.7 ± 7.7
Male	55 (76%)	53 (74%)
Female	17 (24%)	19 (26%)
Causes of ARDS		
SARS-CoV2	52 (72%)	49 (68%)
Aspiration	3 (4%)	4 (6%)
Viral pneumonia (HSV etc.)	2 (3%)	1(1%)
Bacterial pneumonia	1 (1%)	5 (7%)
Sepsis	6 (8%)	4 (6%)
Pancreatitis	2 (3%)	1 (1%)
Thoracic trauma	1 (1%)	2 (3%)
Other	5 (7%)	6 (8%)
Comorbidities, no. (%)		
Hypertension	37 (51%)	33 (46%)
Unknown	4 (6%)	3 (4%)
Diabetes	24 (33%)	17 (24%)
COPD	1 (1%)	10 (14%)**
OSAS	4 (6%)	3 (4%)
Asthma	5 (7%)	2 (3%)
Sarcoidosis	1 (1%)	0 (0%)
Emphysema	0 (0%)	4 (6%)***
Interstitial lung disease	0 (0%)	1 (1%)
Tumor	1 (1%)	3 (4%)
LAE	1 (1%)	2 (3%)**
Chronic kidney disease (GFR < 60)	5 (7%)	5 (7%)
Cardiac disease	11 (15%)	16 (22%)
Obesity	12 (17%)	12 (17%)
Transplantation	2 (3%)	0 (0%)
HIV	1 (1%)	0 (0%)
Immune suppression	5 (7%)	2 (3%)
Psychiatric diseases	4 (6%)	12 (17%)*
Neurological diseases	11 (15%)	7 (10%)
Liver disease	5 (7%)	3 (4%)
Coagulopathy	0 (0%)	3 (4%)
Tumor (anamnestic)	2 (3%)	7 (10%)
OSAS	4 (6%)	3 (4%)**
SOFA admission score, mean ± SD^b^	10.8 ± 3.2	10.8 ± 3.7
Reasons for ICU admission		
Medical	62 (86%)	60 (83%)
Surgery	4 (6%)	2 (3%)
Emergency surgery	6 (8.3%)	10 (14%)
Treatments used in COVID-19 patients		
Steroids*	43 (68.3%)	31 (50.8%)
IL-6 antibodies*	12 (19.0%)	11 (18.0%)
Remdesivir*	37 (58.7%)	24 (39.3%)

^a^142 patients included; ^b^135 patients included, *124 patients included

*p = 0.034, **p = 0.005, ***p = 0.043

### Primary outcome

**Fig. 2:**
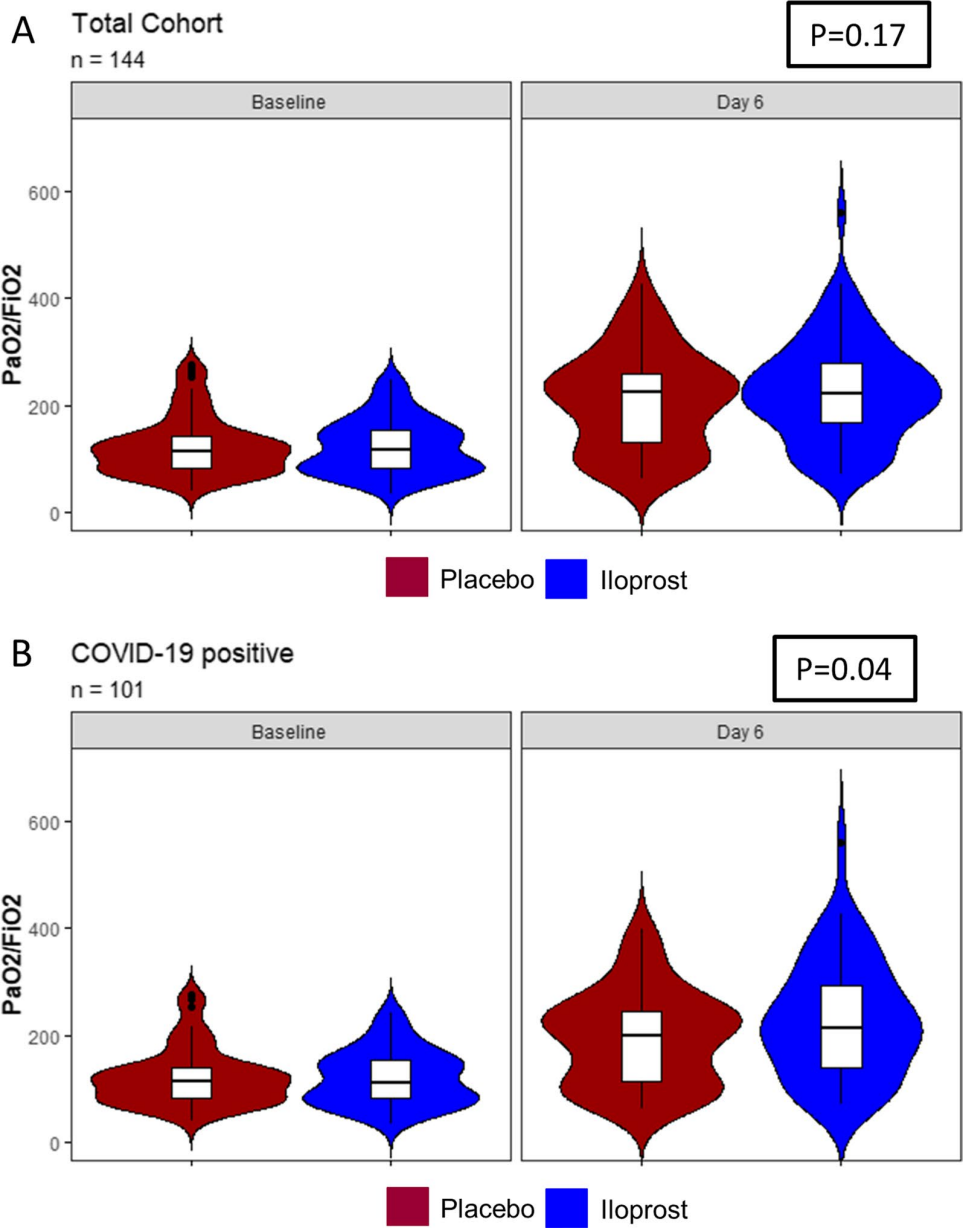
PaO_2_/FiO_2_ ratio in the total study cohort and COVID-19 positive patients

We defined the PaO_2_/FiO_2_ ratio on Day 5 following treatment with the study drug as the primary outcome, and the PaO_2_/FiO_2_ ratio at baseline was not significantly different between groups. Following treatment with prostacyclin, the PaO_2_/FiO_2_ ratio showed a tendency to improve when considering all patients included in the trial (Fig. 1 and Additional file 1: Fig. S1A–E). Therefore, the primary group showed a tendency toward improvement (difference in improvement prostacyclin vs. placebo groups of 19.5 mmHg, baseline adjusted 20.1 mmHg, p = 0.177, 95% CI (− 9.1)–(+ 49.4)) following prostacyclin inhalation (Table 2, Fig. 2). The interaction between the baseline and treatment arm was not significant (p = 0.94). In addition, the interaction between ventilatory ratio (VR) in the Placebo and the treatment arm was also not significant (p = 0.97, Additional file 1: Fig. S2, Additional file 1: Table S3). Sex (p = 0.073, female vs. male 33.4 mmHg), age (0.11 mmHg per year, p = 0.85), direct vs. indirect injury (indirect vs. direct injury 58.8 mmHg, p = 0.068), or COVID (no COVID vs. COVID 28.0 mmHg p = 0.115) and ventilatory ratio (p = 0.061) were not prognostic factors; however, differences might be relevant for each factor except for age (Additional file 1: Table S4).

**Table 2:** Main clinical outcomes

	Control (n = 72)	Prostacyclin (n = 72)	p-value
PaO_2_/FiO_2_ ratio			
Baseline	123.6 ± 54.0 (111.0–136.2)	123.2 ± 51.0 (11.3–135.0)	0.96
Day 5	208.6 ± 92.1 (186.9–230.4)	227.9 ± 97.5 (204.7–251.1)	0.24
Difference Day 5—Baseline^a^	85.0 ± 84.3 (65.0–105.0)	104.7 ± 90.5 83.1–126.3)	0.189*
Death at 90 days	22 (31%, 20–42%)	23 (32%, 21–44%)	
SOFA at day 7^c^	9.0 ± 4.7 (7.7–10.3)	8.6 ± 4.7 (7.3–9.9)	
SOFA at day 14^d^	9.7 ± 5.7 (7.7–11.8)	10.5 ± 5.1 (8.7–12.3)	
SOFA at day 28^e^	10.8 ± 5.7 (7.1–14.4)	8.8 ± 5.6 (5.6–12.0)	
Duration of ventilation			
Including pauses in d	11 (11–14, 8–14)	11 (7–14, 9–14)	
ICU length of stay in d	16 (10–34, 14–23)	17 (12–43, 14–28)	
Ventilator associated pneumonia^f^	5 (7%, 2–15%)	5 (7%, 2–16%)	
ICU acquired weakness^g^	7 (10%, 4–19%)	4 (6%, 2–14%)	
Discharge location^h^			
Home	20 (41%, 27–58%)	19 (40%, 26–55%)	
Skilled nursing facility	1 (2%, 0–11%)	1 (2%, > 0–11%)	
Rehabilitation unit	3 (6%, 1–17%)	6 (13%, 5–25%)	
Other transfer unit	25 (51%, 36–66%)	22 (46%, 31–61%)	

^a^142 patients included; ^c^109 patients included; ^d^65 patients included; ^e^26 patients included; ^f^143 patients included; ^g^140 patients included; ^h^97 patients included *p-value differs from baseline adjusted analysis (p = 0.177), Entries are mean ± SD, median interquartile range or absolute and percentage frequency, results in brackets are 95% CIs for the mean or Interquartile ranges and 95% CIs for the median or 95% CIs for proportions. Death at 90 days RR = 1.05 (95% CI 0.93–1.18), Risk difference = 1.4% (95% CI (− 13.8%)–(+ 16.5%)

Additional file 1: Fig. S3 shows the results for the primary outcome stratified by subgroups. When examining the subset of patients with COVID-19-induced ARDS, we observed a significant increase in the PaO_2_/FiO_2_ ratio on Day 5 in patients treated with prostacyclin compared to patients with placebo (34.4 mmHg, p = 0.043). The interaction between COVID-19 and treatment was not significant (p = 0.104). For additional details, see Fig. 2. Treatment effects were comparable for male patients (16.7 mmHg, p = 0.28) and the smaller subgroup of female patients (25.6 mmHg, p = 0.49). A trend toward a larger treatment effect on elderly patients was observed, increasing from patients aged 20 to 39 years (− 4.7 mmHg, in favor of the control, p = 0.85) to 24.4 mmHg in patients aged 70 years or older (24.4 mmHg, p = 0.45). However, the interaction between age and treatment was not significant (p = 0.28). The effect on patients with direct injury was considerably larger (24.6 mmHg, p = 0.107) than that on the very small group of patients with indirect lung injury (− 80.4 mmHg in favor of the control, p = 0.077). The interaction was significant (p = 0.029).

### Secondary outcomes

**Fig. 3:**
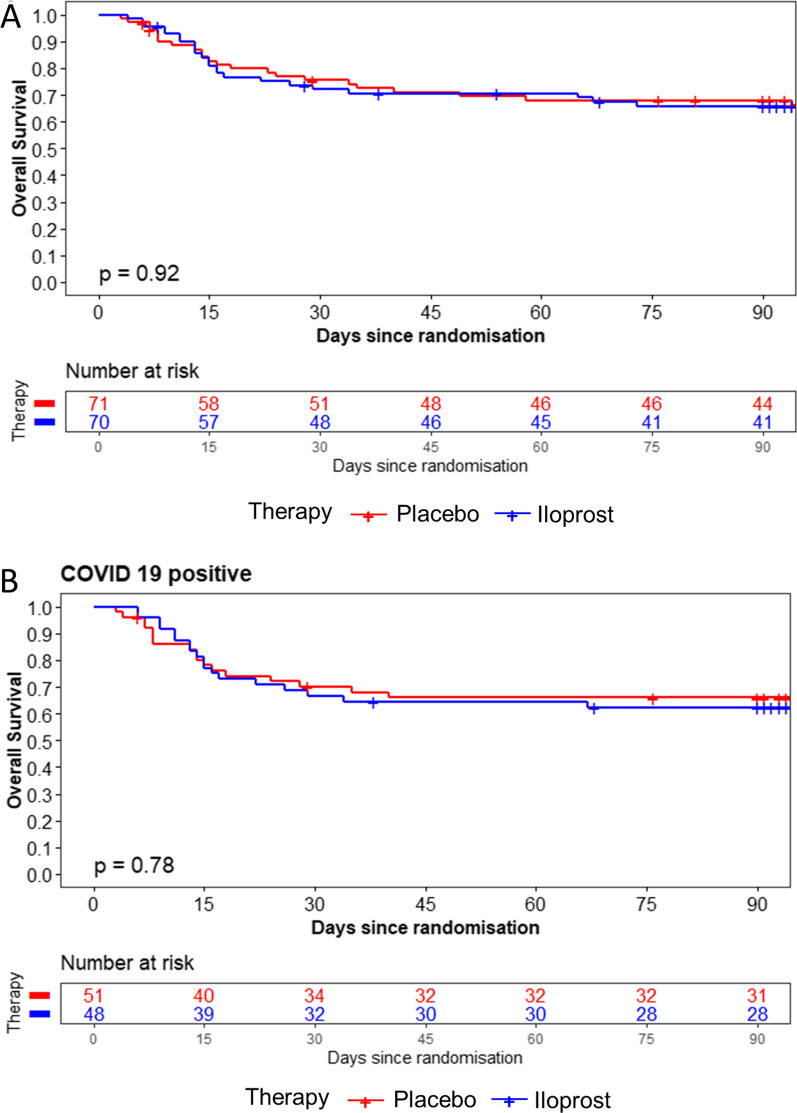
Overall survival in the total study cohort and COVID-19 positive patients

Secondary outcomes were not significantly different between groups. Following treatment with prostacyclin, the mortality rate did not improve when analyzing all patients with ARDS (Fig. 3). Regarding survival, no treatment differences were observed in any subgroup (p > 0.4) in either male or female patients, in any age stratum, in patients with direct or indirect lung injury or in patients with or without COVID-19 (Fig. 3 and Additional file 1: Fig. S4). In the total sample, no difference in the SOFA scores on Days 7, 14 and 28 were observed between study arms. The duration of mechanical ventilation and ICU length of stay did not differ between groups. The incidence of ventilator-associated pneumonia and ICU acquired weakness also did not differ between groups. The discharge location was also similar in both groups (Table 2).

When analyzing the subset of patients with COVID-19, we found that the secondary outcomes were not significantly different between groups. In this subgroup of patients, treatment with prostacyclin did not improve secondary outcomes. The SOFA score of patients with COVID-19 was not improved on Days 7, 14 and 28. The duration of mechanical ventilation and ICU length of stay did not differ between groups of patients with COVID-19. The incidence of ventilator-associated pneumonia, discharge location and ICU-acquired weakness also did not change in patients with COVID-19 following treatment with prostacyclin.

### Adverse events

Adverse events did not differ significantly between groups. In the treatment group, we identified a similar incidence of bleeding complications than in the placebo group (9 vs. 11). Similar results were also obtained for the transfusion requirements. The incidence of thrombotic pulmonary embolism, coagulopathy, need for RRT and incidence of gastrointestinal complications also did not differ between groups. Neurological and cardiovascular complications were similar in both groups (Table 3).

**Table 3:** Adverse events

	Control	Prostacyclin
Bleeding, no. (%)	11 (15%0.8–26%)	9 (13%, 6–22%)
Transfusion requirement (RBC), no. (%)^i^	24 (34%, 23–46%)	24 (34%, 23–46%)
Thrombotic event, pulmonary embolism or coagulopathy	5 (7%, 2–15%)	5 (7%, 2–15%)
Need for renal replacement therapy	17 (24%, 14–35%)	15 (21%, 12–32%)
Gastrointestinal complications, no. (%)	13 (18%, 10–29%)	7 (9%, 4–19%)
Neurologic complications, no. (%)	2 (3%, 0.3–10%)	4 (6%, 2–14%)
Cardiovascular complications, no. (%)	17 (24%, 14–35%)	13 (18%, 10–29%)

^i^142 patients included, results in brackets are 95% CIs for proportions

In patients with COVID-19, the incidence of adverse events was not significantly different between groups. We observed the same incidence of bleeding complications in the treatment group and the placebo group. Similar results were obtained for the transfusion requirements. The incidence of thrombotic pulmonary embolism, coagulopathy, need for RRT and incidence of gastrointestinal complications also did not differ between groups. The incidences of neurological and cardiovascular were similar in both groups.

## Discussion

In this randomized controlled trial involving patients with ARDS, we addressed the question of whether inhaled prostacyclin would improve the lung function, as measured by oxygenation in the blood. We were able to show improved oxygenation on Day 5 of treatment in a population with ARDS however, the effect was not significant. The observed effect of prostacyclin was not associated with improved secondary outcomes in the intervention group, and neither the overall outcome nor the incidence of secondary complications was significantly different between groups.

In addition to extensive inflammation within the alveolar space, the central hallmark of ARDS is hypoxia [13, 14]. Prone positioning and the use of extracorporeal membrane oxygenation (ECMO) have been shown to reduce hypoxia and to increase oxygenation [15, 16]. ECMO therapy, however, is limited to expert centers and cannot be used widespread in all hospitals caring for these patients, since it involves a significant logistical effort and expert knowledge. Therefore, pharmaceutical approaches to improve pulmonary function are still very important. We described in this trial that a prostacyclin intervention only showed a non-significant tendency toward exerting a positive effect on oxygenation in critically ill patients with ARDS. In a small case study of twenty patients, Sawheny et al. showed that oxygenation in patients with ARDS was improved by administering inhaled prostacyclin [9]. Johanssen et al. showed that the intravenous administration of prostacyclin in COVID-19 ARDS with endotheliopathy was not associated with a significant reduction of mortality, but a point estimate analysis done after the end of the trial favored the prostacyclin group [10]. However, these two studies performed were either done without a control group, did not employ a randomized prospective design or used a different administration strategy for prostacyclin. Therefore, data about the role of prostacyclin acquired with a RCT design in patients with ARDS are still sparse to date.

As mentioned above, this randomized study documents the effect of prostacyclin on patients with ARDS including COVID-19-induced ARDS. COVID-19-induced ARDS is an entity characterized by additional features compared to classical ARDS. Patients with COVID-19 present widespread pulmonary microthrombi and inflammatory infiltrates with diffuse pulmonary fibrosis [8, 17]. In addition, endothelial dysfunction and a severe inflammatory response are indicators of COVID-19-induced pulmonary failure. Furthermore, hypoxemia that is unrelated to lung mechanics is present in patients with COVID-19-induced ARDS [18]. These pathological features are patterns that could be influenced by prostacyclin. Prostacyclin controls platelet aggregation and aggregability, preventing thrombus formation in an environment with a damaged endothelium [19, 20]. In addition, prostacyclin interacts with and enhances the effect of nitric oxide on the vascular surface [21]. As a result, endothelial function is improved, microthrombi are prevented, and the inflammatory response is reduced by administering prostacyclin to these patients. All of the described effects could have beneficial functions in patients with ARDS, especially in patients with COVID-19-induced ARDS.

Of course, our trial also has several limitations. First, the trial was started before the COVID-19 pandemic to evaluate the effects of prostacyclin on oxygenation and outcomes of critically ill patients with ARDS. Then, shortly after the start of the trial, the first wave of patients with COVID-19-induced ARDS were treated in Germany and German ICUs, including ours. Given the potential differences in the pathologies of ARDS and COVID-19-induced ARDS, this factor might have significant implications for therapy with prostacyclin. However, we decided to include all patient groups with ARDS and not exclude patients with COVID-19, since our trial should also take advantage of the opportunity to compare patients with different ARDS etiologies and their responses to prostacyclin treatment. Second, our sample size was moderate, and our study was probably underpowered. This interpretation seems justified, as we obtained the expected effect, i.e., a superiority of 21 mmHg in PaO_2_/FiO_2_, but the standard deviations were much larger, as expected (80 mmHg in the controls, 91 mmHg in the prostacyclin group vs. 40 mmHg assumed). Third, the intervention group and the control group differed significantly in age, which could have a potential effect on the overall outcome in this patient group. The average age was older in the intervention group, and therefore, one would expect this factor to have a potential negative effect if any effect at all, based on the literature [22, 23]. However, in our sample, no significant association of age with the primary outcome was observed. We also included patients receiving ECMO in this trial, which is particularly important because we measured oxygenation as the primary outcome. We recorded a nonsignificant difference between 21 patients treated with ECMO in the control group and 14 patients treated with ECMO in the treatment group, but of course, ECMO is important for the oxygenation levels measured. This is remarkable since the larger number in the control group would potentially skew the oxygenation toward the control group on Day 6, but we did not observe this result. The treatment groups still performed better when analyzing the primary outcome oxygenation and supported the positive effect of prostacyclin on oxygenation. Fourth, although the study medication assignment was randomized, we did not blind the investigators to the study medication, which was not possible due to the complex nature of the preparation of the prostacyclin in a blinded manner in our setting; therefore, we did not pursue this approach. Fifth, we included patients who had ARDS due to multiple reasons, and patients with and without COVID-19. However, impaired oxygenation is the common cardinal symptom of patients with all forms of ARDS, and most clinical approaches to improve oxygenation in all patients were tested in heterogeneous clinical ARDS groups, since we wanted to identify a commonly used intervention that would improve the poor oxygenation status. Therefore, we included all patients who met the inclusion criteria.

In conclusion, among patients with severe ARDS, inhaled prostacyclin showed a tendency to improve oxygenation, especially in COVID-19-induced ARDS. This change was not associated with a survival benefit but was associated with an improvement of secondary outcomes in the treated patient population. Larger clinical trials will evaluate the effect of prostacyclin on the overall outcomes of patients with ARDS.

## Supplementary Information

**Additional file 1: Table S1.** Organ Specific Baseline Characteristics and Ventilation Parameters. **Table S2.** Primary Endpoint, COVID 19 patients only. **Table S3.** Primary Endpoint (PaO_2_/FiO_2_ ratio Day 5), adjusted by Ventilatory ratio and therapy. **Table S4.** Primary Endpoint in age strata. **Figure S1.** A) PaO_2_/FiO_2_ (min) during follow up with Iloprost vs placebo, B) PaO_2_/FiO_2_ (max) during follow up with Iloprost vs placebo. C) PaO_2_/FiO_2_ before and during therapy with Iloprost vs placebo total cohort, D) Covid-19 Positive, E) Covid-19 negative. **Figure S2.** Ventilatory ratio (VR) in the Placebo and treatment arm. **Figure S3.** A) Ninety-day mortality rates in COVID-19-negative and COVID-19-positive patients and B) 90-day mortality rates in COVID-19-negative patients in the Iloprost-treated group compared with control (NaCl)-treated patients. **Figure S4.** Subgroup analysis.

## Data Availability

After publication, the data will be made available to others on reasonable requests to the corresponding author. A proposal with detailed description of study objectives and statistical analysis plan will be needed for evaluation of the reasonability of requests. Additional materials might also be required during the process of evaluation. Data will be provided after approval from the University of Tübingen.
