# Supplementary material for: Inhaled prostacyclin therapy in the acute respiratory distress syndrome: a randomized controlled multicenter trial
Source: Respir Res. 2023 Feb 18;24:58. doi: 10.1186/s12931-023-02346-0 (PMC9938510; doi:10.1186/s12931-023-02346-0)
Supplement: Supplementary file 1 — Additional file 1: Table S1. Organ Specific Baseline Characteristics and Ventilation Parameters. Table S2. Primary Endpoint, COVID 19 patients only. Table S3. Primary Endpoint (PaO2/FiO2 ratio Day 5), adjusted by Ventilatory ratio and therapy. Table S4. Primary Endpoint in age strata. Figure S1. A) PaO2/FiO2 (min) during follow up with Iloprost vs placebo, B) PaO2/FiO2 (max) during follow up with Iloprost vs placebo. C) PaO2/FiO2 before and during therapy with Iloprost vs placebo total cohort, D) Covid-19 Positive, E) Covid-19 negative. Figure S2. Ventilatory ratio (VR) in the Placebo and treatment arm. Figure S3. A) Ninety-day mortality rates in COVID-19-negative and COVID-19-positive patients and B) 90-day mortality rates in COVID-19-negative patients in the Iloprost-treated group compared with control (NaCl)-treated patients. Figure S4. Subgroup analysis. [file 12931_2023_2346_MOESM1_ESM.docx]

**SUPPLEMENTAL DATA**

**Inhaled Prostacyclin Improves Oxygenation in Patients with COVID-19-induced Acute Respiratory Distress Syndrome (ARDS) but not in other etiologies:**

**a prospective randomized controlled multicenter trial (Thilo trial)**

Helene A. Haeberle, Stefanie Calov, Peter Martus, Lina Maria Serna-Higuita, Michael Koeppen, Almuth Goll, Alice Marie Bernard, Alexander Zarbock, Melanie Meersch, Raphael Weiss, Martin Mehrländer, Gernot Marx, Christian Putensen, Tamam Bakchoul, Harry Magunia, Bernhard Nieswandt, Valbona Mirakaj^#^ and Peter Rosenberger^#^

**Methods**

**Study design, Ethics and Oversight**

A data safety and monitoring board oversaw the study and reviewed safety data periodically. Onsite monitoring for correctness of the consent procedure was performed at all sites by the Center for Clinical Studies Tübingen (Germany). The members of the writing committee wrote all drafts of the manuscript. All authors approved the final version of the manuscript and made the decision to submit it for publication.

**Patients inclusion and exclusion criteria**

Patients received a study identification number and a treatment allocation at enrollment if inclusion criteria were met.

Inclusion criteria were: 1) PaO_2_/FiO_2_ ≤ 300 at time of ARDS diagnosis, 2) bilateral opacities on a frontal chest radiograph, 3) required positive pressure ventilation via an endotracheal tube or noninvasive ventilation, 4) no clinical signs of left atrial hypertension, and 5.) an “acute onset” defined as a duration of the hypoxemia criterion (#i) and the chest radiograph criterion (#ii) ≤ 28 days at the time of randomization. Patients were required to be randomized within 96 hours of the ARDS diagnosis and no later than 7 days from the initiation of mechanical ventilation.

Patients were excluded if: 1) the patient, surrogate or physician was not committed to full intensive care support, 2) patients had a positive pregnancy test at the time of screening, 3) had contraindications for the use of prostacyclin 4) received nitric oxide or prostacyclin therapy within the previous 24 h before study randomization, or 5) were dependent on the sponsor, investigator, manufacturer and/or their employees.

**Sample size**

In our study, we assumed an effect size of 0.525, leading to 116 error degrees of freedom to achieve a power of 80% using a level of significance of 0.05 in the two-sided t-test. The interindividual effect size of 0.525 combined with the standard deviation of 40 corresponds to a difference of approximately 21 in the p_a_O_2_/FiO_2_ ratio in treatment compared to controls. Therefore, we calculated numbers of patients to be assessed for eligibility (n=300), assigned to the trial (n = 150), and analyzed (n=150 in the intention-to-treat analysis). The sample size and power consideration referred to 120 evaluable patients.

**Statistical analysis**

The primary endpoint, P_a_O_2_/FiO_2_, on Day 6 after baseline, was evaluated using a baseline-adjusted analysis of covariance model with the last measurement of p_a_O_2_/FiO_2_ before treatment serving as the baseline and the study arm and center as two-level factors. Additionally, the interaction between baseline and treatment was tested and included in the model if the result was significant. In the case of an interaction, the main effect was retrieved for the arithmetic mean of baseline values using the centered variable for p_a_O_2_/FiO_2_. Multiple imputation was applied. The level of significance was 0.05 (two-sided), and no interim analysis was performed. Only the primary analysis was confirmatory. Subgroup analyses were planned for sex and race, patients with increased pulmonary arterial pressure, direct or indirect lung injury, and age by decades, but only in groups with 40 patients or more. In the final analysis, we combined age groups, and we also report results from strata of sizes slightly smaller than 40. An additional primarily unplanned subgroup analysis was performed for patients with COVID, as the pandemic only occurred during the course of the study. The same factors were also tested for prognostic value.

Finally, a secondary analysis was performed including the ventilatory ratio (VR) at baseline, as an indicator of impaired efficiency of ventilation. VR was calculated using the following equation (ref: DOI: 10.1164/rccm.201804-0692OC):

VR = $\frac{VE measured X PaCo2 measured}{VE predicted X PaCO2 ideal}$

where VE_measured_ was the measured minute ventilation (ml/min), VE_predicted_ was the predicted minute ventilation calculated as predicted body weight*100 (ml/min), predicted body weight was calculated using the NHLBI ARDS network formula (female = 45.5 + 0.91*(height in cm – 152.4), male = 50 + 0.91*(height in cm – 152.4), and Pa_CO2ideal_ was set as 37.5 mm Hg for all patients^1^.

The statistical analysis of the prespecified secondary endpoints was performed with descriptive and exploratory statistical methods according to the scale and observed distribution. P values were reported, although all secondary analyses are nonconfirmatory.

**SUPPLEMENTAL TABLES**

| Supplemental Table 1: Organ Specific Baseline Characteristics and Ventilation Parameters | | |
| --- | --- | --- |
|  | **Control (n=72)** | **Prostacyclin (n=72)** |
|  |  |  |
| ECMO yes/ no ^i^ | 21 (29%) | 15 (21%)^m^ |
| COVID-19 + ECMO | 17 (23%) | 14 (19%) |
| Duration of ECMO (days), median (IQR) | 17 (11-38) | 27 (9-55) |
| Inspiratory Plateau Pressure cmH2O median (IQR) | 20 (18-23) | 20 (17-23) |
| Tidal Volume/ kg predicted body weight | 6.3 ±2.5 | 6.7 ±1.9 |
| Driving Pressure cmH_2_O | 12 (9-15) | 13 (11-16) |
| Acidosis | 24 (34%) | 36 (50%)* |
| Lactate level (Minimum), Median (IQR) mmol/l | 1.00 (0.80-1.20) | 0.95 (0.70-1.38) |
| Lactate level (Maximum), Median (IQR)  mmol/l | 1.40 (1.13-1.80) | 1.5 (1.10-2.18) |
| Alananine Aminotransferase (U/l) ALT ^j^ , median (IQR) | 34 (22-54) | 35 (24-68) |
| Aspartate Aminotransferase (U/l) AST ^k^ | 48 (32-64) | 59 (37-96) |
| INR | 1.2 (1.1-1.6) | 1.2 (1.1-1.5) |
| Bilirubin (Median, IQR) | 0.7 (0.4-1.2) | 0.6 (0.4-1.1) |
| Creatine Kinase (U/l) | 287 (112-768) | 261 (75-730) |
| BUN, median (IQR) ^l^ | 46 (30-64) | 52 (35-68) |
|  |  |  |
| Hemoglobin level (g/l) | 10.0 ± 2.3 | 10.4 ± 2.2 |
| Platelet Count (nx10^3^/µl) | 231 (167-300) | 207 (144-307) |
|  |  |  |
| Vasopressor Dependent No | 62 (87%) | 61 (85%) |
| Lowest Mean Arterial Blood Pressure mmHg | 65.1 ± 8.9 | 64.2 ± 7.6 |

Entries are mean ± SD, results in brackets are 95% CIs for the mean, i= 142 patients included; j= 119 patients included; k= 117 patients included; l= 143 patients included^m^p = 0.05

**Supplemental Table 2 Primary Endpoint, COVID 19 patients only (imputated data, n=101 each analysis)**

| PaO2/FiO2 ratio | Control | Prostacyclin | p-value |
| --- | --- | --- | --- |
| Baseline | 121.6 ± 53.3 (107.0-136.3 | 121.0 ± 50.6 (106.6-135.3) | 0.95 |
| Day 5 | 193.5 ± 88.4 (168.9-218.2) | 227.3 ± 109.0 (196.1-258.4) | 0.093 |
| Difference Day 5 - Baseline^a^ | 71.9 ± 78.0 (50.0-93.7) | 106.3 ± 96.5 (78.7-134.0) | 0.054* |

Entries are mean ± SD, results in brackets are 95% CIs for the mean, ^*^p value differs from baseline adjusted analysis (p=0.043)

**Supplemental Table 3 Primary Endpoint (PaO2/FiO2 ratio Day 5), adjusted by Ventilatory ratio and therapy (analysis of covariance)**

| PaO2/FiO2 ratio | df | F | Beta | 95% CI | p-value | Eta Quadrat |
| --- | --- | --- | --- | --- | --- | --- |
| Intercept |  |  | 88.26 | 32.67 ; 143.86 |  |  |
| Therapy Prostacyclin | 1 | 1.72 | 21.63 | 11.07 ; 54.32 | 0.19 | 0.015 |
| Ventilatory ratio | 1 | 0.43 | 10.08 | -20.3 ; 40.48 | 0.51 | 0.004 |

**Supplemental Table 4: Primary Endpoint in age strata (imputed data, n=144 in each analysis**)

| PaO2/FiO2 ratio | Control | Prostacyclin |
| --- | --- | --- |
| 20-<40 ys |  |  |
| Baseline | 123.9 ± 73.5 | 121.7 ± 49.6 |
| Day 5 | 248.0 ± 135.4 | 245.2 ± 97.5 |
| Difference Day 5 - Baseline | 124.1 ± 109.4 | 123.5 ± 98.1 |
| 40-<60 ys |  |  |
| Baseline | 110.2 ± 46.8 | 113.6 ± 123.3 |
| Day 5 | 191.8 ± 92.4 | 211.6 ± 53.1 |
| Difference Day 5 - Baseline | 81.5 ± 86.5 | 98.0 ± 109.4 |
| 60-<70 ys |  |  |
| Baseline | 133.4 ± 48.3 | 120.0 ± 43.5 |
| Day 5 | 211.4 ± 67.8 | 221.2 ± 87.2 |
| Difference Day 5 - Baseline | 78.0 ± 66.5 | 101.2 ± 77.9 |
| 70ys and older |  |  |
| Baseline | 154.5 ± 52.8 | 137.8 ± 53.7 |
| Day 5 | 229.0 ± 63.1 | 247.4 ± 68.0 |
| Difference Day 5 - Baseline | 74.5 ± 71.0 | 109.6 ± 76.1 |

Entries are mean ± SD, 20-<40 ys n=16, 40-<60 ys n=56, 60-<70 ys n=44, 70+ ys n=38 All p-values were larger than 0.4

1. Sinha P, Calfee CS, Beitler JR, et al. Physiologic Analysis and Clinical Performance of the Ventilatory Ratio in Acute Respiratory Distress Syndrome. *Am J Respir Crit Care Med.* 2019;199(3):333-341.
